# The Application of DNA Viruses to Biotechnology

**DOI:** 10.3390/v17030414

**Published:** 2025-03-14

**Authors:** Adam J. Schieferecke, Nadia Kuxhausen Ralph, David V. Schaffer

**Affiliations:** 1 Department of Molecular & Cell Biology, University of California, Berkeley, Berkeley, CA 94720, USA; nadiakuxralph@berkeley.edu (N.K.R.); schaffer@berkeley.edu (D.V.S.); 2 California Institute for Quantitative Biosciences, University of California, Berkeley, Berkeley, CA 94720, USA; 3 Department of Chemical and Biomolecular Engineering, University of California, Berkeley, Berkeley, CA 94720, USA; 4 Department of Bioengineering, University of California, Berkeley, Berkeley, CA 94720, USA; 5 Helen Wills Neuroscience Institute, University of California, Berkeley, Berkeley, CA 94720, USA

**Keywords:** gene therapy, oncolytic virotherapy, vaccine, agriculture, phage therapy, microbiome editing, adeno-associated virus, vaccinia virus, adenovirus, herpesvirus

## Abstract

The delivery of biomolecules to target cells has been a longstanding challenge in biotechnology. DNA viruses naturally evolved the ability to deliver genetic material to cells and modulate cellular processes. As such, they inherently possess requisite characteristics that have led to their extensive study, engineering, and development as biotechnological tools. Here, we overview the application of DNA viruses to biotechnology, with specific implications in basic research, health, biomanufacturing, and agriculture. For each application, we review how an increasing understanding of virology and technological methods to genetically manipulate DNA viruses has enabled advances in these fields. Additionally, we highlight the remaining challenges to unlocking the full biotechnological potential of DNA viral technologies. Finally, we discuss the importance of balancing continued technological progress with ethical and biosafety considerations.

## 1. Introduction

The ability to deliver genetic cargoes into targeted cell types is central to the success of many biotechnological approaches, and the past 15 years have brought remarkable advances in genetically encodable biotechnologies [1,2,3]. Viruses evolved over millions of years to deliver nucleic acids and proteins to cells. Accordingly, researchers have co-opted some viral species for use in applications ranging from the vaccination against pathogenic microbes, the treatment of genetic diseases, the selective elimination of cancer, protein manufacturing, and pest control. Furthermore, applications to combat bacterial antibiotic resistance, develop agricultural crops and livestock with improved health content and resilience to changing climates, and engineer the human microbiome for health benefits represent emerging uses of viral-based gene delivery systems. Here, the applied biotechnological uses of DNA genome-containing virus vectors are outlined. Common themes among all applications, as well as limitations and remaining challenges, are discussed. Finally, the importance of continued research and development of technologies to engineer DNA viruses as delivery vectors to solve scientific, agricultural, environmental, manufacturing, and health problems is advocated, while ethical and biosafety considerations are considered.

## 2. Main

### 2.1. Vaccination

Vaccines that are based on attenuated DNA viruses have been historically instrumental in controlling dangerous human and animal pathogens (Table 1). For example, the vaccinia virus (VACV) is attenuated in humans but shares significant antigenic overlap with pathogenic members of the *Orthopoxvirus* genus, such as monkeypox virus (the causative agent of mpox disease) and variola virus (the causative agent of smallpox disease) [4,5,6]. This quality led to the use of live, replication-competent VACV as a protective vaccine against orthopoxviruses, resulting in smallpox being the first and only human pathogen to be successfully eradicated to date [7,8]. As of January 2025, two VACV-based vaccines are approved in the United States (US) by the Food and Drug Administration (FDA) for clinical use: (1) ACAM2000, which is based on the replication-competent New York Board of Health VACV strain [9,10] and (2) Jynneos, which is based on the non-replication-competent modified vaccinia Ankara (MVA) strain [11,12]. These live DNA virus vaccines were crucial to containing the global mpox outbreak of 2022 and continue to protect laboratory, military, and medical personnel who have occupational exposure to orthopoxviruses [13]. A second example of clinically approved vaccines based on DNA viruses includes the use of a weakened form of a herpesvirus known as varicella-zoster virus (VZV) to prevent chickenpox and/or shingles, which are highly contagious diseases caused by wild-type VZV [14]. As of January 2025, there are three FDA-approved vaccines utilizing live attenuated herpesviruses that have been shown to safely and effectively prevent these conditions: (1) Varivax [15,16], (2) Proquad [17,18], and (3) Zostavax [19,20,21]. As a result of the widespread use of these vaccine products, Chickenpox, which was previously common in the US, is now rare [22]. A third example of a successful vaccine based on a DNA virus is the “Adenovirus Type 4 and Type 7 Vaccine, Live, Oral”, which is licensed for clinical use in the US but currently administered only to military personnel [23].

In addition to the use of attenuated DNA virus-based vaccines to elicit protective immunity against antigenically similar pathogens, recombinant versions of DNA viruses have been used as vectored vaccines to protect against antigenically distinct pathogens. For example, a recombinant VACV expressing the Rabies glycoprotein is approved for use in animals [24]. In addition, recombinant adenovirus (AdV) vaccines encoding the SARS-CoV-2 spike protein received emergency authorization during the COVID-19 pandemic in the United Kingdom (ChAdOx1 nCoV-19/AZD1222 developed by the University of Oxford partnered with the private company AstraZeneca) [25,26], US (Ad26.COV2.S developed by the private company Janssen Biotech, Inc., now named Johnson & Johnson Innovative Medicine) [27,28], and dozens of other countries. Moreover, recombinant AdV and VACV vaccine platforms are the subject of hundreds of recent pre-clinical studies [29,30,31,32,33,34,35] and ongoing clinical trials [36,37,38,39]. Additional vaccination platforms based on recombinant DNA viruses, including AAVs [40,41,42,43,44], insect viruses [45,46,47,48,49,50,51], and heterologous vaccines consisting of two or more viral vectors delivered simultaneously [52,53,54], are also under pre-clinical and clinical investigation.

There are several theoretical advantages of using DNA viral vectors over other approaches to deliver antigens. First, DNA viruses have generally shown higher in vivo delivery efficiencies to target cells, leading to increased expression levels of antigens of interest. Second, whole DNA viruses serve as adjuvants and are thus capable of more realistically emulating natural infection compared, for example, to recombinant protein or mRNA vaccines. Third, DNA viruses are more stable than mRNA and can be lyophilized for transport, thus increasing accessibility [55,56]. On the other hand, whole DNA viruses can be more challenging to manufacture than naked DNA, mRNA, or protein subunits. Recombinant DNA virus platforms also may contain epitopes that possess immunodominance (due to high affinities for MHC/HLA) over the heterologous antigen of interest, potentially affecting immune tolerance for the heterologous antigen of interest [57,58,59].

Human health will continue to be threatened by both existing pathogens and future pathogens emerging from natural spillover events or potential acts of bioterrorism. Aided by further advances in virology, next-generation vaccines based on DNA viral vectors have strong potential to help contain and eradicate pathogens.

### 2.2. Gene Therapy

Gene therapy, defined as the delivery of nucleic acids to cells for the expression of therapeutic molecules to treat disease, is a biotechnological application for which DNA viral vectors are uniquely enabling. In the past several decades, gene therapy has moved from a concept to a viable standard-of-care treatment for an increasing number of monogenic diseases. Stable gene expression mediated by engineered versions of the single-stranded DNA (ssDNA) parvovirus known as adeno-associated virus (AAV) has been central to the clinical success of in vivo gene therapies to date. As of January 2025, seven AAV-based viral vectors that facilitate the addition of genes for long-term expression of therapeutic proteins have been approved by global regulators for clinical use in humans (Table 2): voretigene neparvovec-rzyl (LUXTURNA), which delivers a functional copy of the normal RPE65 gene to retinal cells to cure an inherited form of vision loss; onasemnogene abeparvovec-xioi (ZOLGENSMA), which replaces the function of the SMN1 gene to treat spinal muscular atrophy (SMA); etranacogene dezaparvovec-drlb (HEMGENIX), which delivers a gene encoding Factor IX to treat hemophilia B; delandistrogene moxeparvovec-rokl (ELEVIDYS), which delivers a gene encoding a shortened form of dystrophin to treat Duchene muscular dystrophy (DMD); valoctocogene roxaparvovec-rvox (ROCTAVIAN), which delivers a gene encoding factor VIII protein to treat hemophilia A; Fidanacogene elaparvovec-dzkt (BEQVEZ), which delivers a functional copy of the Factor IX gene to treat hemophilia B; and eladocagene exuparvovec-tneq (KEBILIDI), which treats aromatic L-amino acid decarboxylase (AADC) deficiency in adults and pediatric patients [60]. In addition, over 110 active- or recruiting-status clinical trials utilizing AAV-based vectors are presently underway [61,62].

The clinical implementation of more complex gene modifications, such as the deletion of deleterious sequences or correction of disease-causing single nucleotide variants, is also possible thanks to recent technological advances in genome editing approaches such as CRISPR-Cas9. However, the high-efficiency delivery of gene editors to targeted cell types in vivo remains a challenge. For example, most CRISPR fusion proteins (e.g., base editors) exceed the limited (~4.7 kb) coding capacity of AAV prior to encoding the gRNA and HDR template sequences. Accordingly, efforts to split cargoes onto multiple recombinant AAV genomes or develop miniaturized promoters and functional gene editors have been underway [63]. In addition to their coding capacity limitations, AAV gene therapy products face manufacturing bottlenecks and immunogenicity challenges, which will need to be addressed by future innovations.

Gene therapy applications that require significantly larger coding capacities [64] can be delivered by nonviral platforms such as lipid nanoparticles (LNPs) or vectors based on large DNA viruses. In general, gene therapy platforms based on LNPs are easier to manufacture than viral gene therapies but suffer from poor biodistribution and insufficient delivery efficiencies to tissues outside of the liver. On the other hand, some large DNA viruses have sufficiently large coding capacities to incorporate HDR repair templates, gRNAs, and proteinaceous components of most CRISPR editing platforms; achieve high entry and nuclear trafficking efficiencies in human cells; and confer long-term expression in targeted cell types. However, large DNA viral vectors are in general more immunogenic than AAVs, which hinders their widespread use in applications that promote the health and longevity of target cells. Despite these limitations, a herpes simplex virus 1 (HSV-1)-based gene therapy called VYJUVEK was recently approved (19 May 2023) by the US FDA. VYJUVEK delivers a functional copy of the COL7A1 gene to treat wounds of patients with dystrophic epidermolysis bullosa [65,66]. With 22 recruiting- and 9 active-phase clinical trials using HSV-based gene therapy approaches currently underway [61], development efforts to deimmunize and improve delivery properties of large DNA viral vectors may unlock the potential of large-cargo gene therapies.

### 2.3. Oncolytic Virotherapy

Despite significant scientific and medical efforts, cancer remains a leading cause of death, killing nearly 10 million people worldwide each year [67]. In contrast to gene therapy applications for which the objective is to promote the health and longevity of target cells, cancer treatment seeks to eliminate target cells. Oncolytic virotherapy (OV) is a special type of vaccine, gene therapy, and immunotherapy [68] that utilizes viruses whose tropism has been directed exclusively to cancer cells. OVs have been widely tested in clinical trials for their capacity to overcome immunosuppressive tumor microenvironments and promote antitumor immunity [69,70].

Among the different OV platforms under consideration, large DNA viral vectors such as those based on AdV, HSV-1, and VACV have shown particular promise due to their clinical safety, genetic deletions resulting in selectivity for tumors, capability to be engineered to enter nearly any human cancer cell type, large transgenic coding capacities, potential to induce long-lasting, tumor-specific immunity, synergism with standard-of-care treatments, and scalable manufacturing processes [71,72,73]. Several factors should be considered when selecting the best OV for a given target tumor. For example, unlike AdV, VACV (1) boasts a cargo coding capacity of >40 kbp, enabling opportunity to encode additional mechanisms of stimulating anti-tumor immune responses within a single vector; (2) utilizes broad entry mechanisms, eliminating the opportunity for cancer cells to evolve resistance at the level of entry; (3) wraps some of its vectors in an outer, exosome-like membrane that is resistant to pre-mature neutralization by complement and antibodies; and (4) undergoes an acute replication cycle in the cytoplasm, which eliminates the risk of persistent expression following treatment.

The idea of using oncolytic viruses as anti-cancer agents dates back to as early as the 19th century when a physician named George Dock noted the remission of tumors in the context of a naturally acquired viral infection [74]. However, because viruses did not evolve to be optimal anti-cancer agents, engineered improvements were needed to develop them as safe and effective treatments. As of January 2025, only a handful of oncolytic virotherapy products have been approved for clinical use in humans: H101 (Oncorine), a replication-competent AdV approved by Chinese regulators in 2005 to treat head and neck cancer; Talimogene laherparepvec (IMLYGIC), a replication-competent oncolytic HSV expressing the granulocyte macrophage colony-stimulating factor (GM-CSF) approved by the US FDA in 2015 to treat melanoma; Teserpaturev (DELYTACT), a G47∆ HSV approved to treat glioblastoma in Japan; and nadofaragene firadenovec-vncg (ADSTILADRIN), a non-replication competent AdV for treatment of high-risk Bacillus Calmette–Guérin (BCG)-unresponsive non-muscle invasive bladder cancer (Table 3). As of January 2025, there are active or recruiting-status clinical trials evaluating 68 AdV vectors, 85 HSV vectors, and 29 VACV vectors to treat a variety of cancers [61]. One notable demonstration of recent clinical momentum is a phase 2 clinical trial (NCT04387461) involving an oncolytic AdV in combination with pembrolizumab to treat BCG-unresponsive non-muscle-invasive bladder cancer, which showed a complete response (CR) rate of 51.4% at 24 months after treatment, surpassing current standard-of-care treatment options [75]. Moreover, recent results from a Ph I clinical trial (NCT03152318) showed that an oncolytic HSV product was effective in treating patients with recurrent glioblastoma, a cancer unresponsive to current immunotherapy [76]. In another example subsequently published by the same group, an oncolytic AdV delivering HSV thymidine kinase, a cargo that serves as a “safety switch” by causing cells to undergo apoptosis in the presence of the prodrug Ganciclovir, was granted Orphan Drug Designation by the FDA to treat pancreatic ductal adenocarcinoma (PDAC) [77]. Alternative large DNA viruses—such as baculoviruses, certain bacteriophages, and myxoma virus—are also under pre-clinical investigation for their potential to deliver therapeutic payloads to human tumors [78,79,80,81].

Despite generally strong safety records and examples of clinical success, OVs based on large DNA viruses require further optimization to achieve their full clinical potential. For example, delivery limitations—which include poor biodistribution to tumors, premature vector neutralization by the immune system, limited intertumoral dissemination, and insufficient intratumoral spread from primary to secondary tumors—have hindered the ability of oncolytic viruses to overcome the exclusion of the adaptive immune system from tumor microenvironments and generate durable immune surveillance against endogenous tumor antigens following treatment. Commonly used immunostimulatory cargoes, such as interferons (IFNs) and certain interleukins (e.g., IL-12), are potent antagonists of viral replication and may thus prevent OVs from optimal amplification in tumors. In addition, the presence of immunogenic viral epitopes and complex interactions between viral and host immunological proteins likely affect the type of immune responses conferred by oncolytic virotherapy treatments. Addressing these issues going forward will further increase the utility of DNA viruses as powerful tools to overcome cancer.

### 2.4. Biomanufacturing

Proteins are manufactured for a variety of purposes, such as the use of enzymes in small-scale molecular cloning to industrial-scale chemical reactions, therapeutic reconstitution of missing proteins into patients, vaccination using protein subunits to protect against infectious diseases, and whole viral gene delivery vectors. Having evolved over millions of years to express exogenous proteins in cells, large DNA viruses have been harnessed to enable large-scale protein production. For example, baculoviruses—a group of nuclear-replicating, large, double-stranded DNA (dsDNA) viruses that exclusively infect insects as host organisms—have been adapted as protein expression systems in insect cells [82,83,84], including commercial cell lines [85,86]. Baculovirus/insectile cell expression systems can produce enormous protein outputs at 27 °C without CO_2_ incubation and have a lower risk of contamination by human pathogens, rendering them advantageous over mammalian expression systems due to increased production efficiency and decreased costs. The baculovirus expression system has also been used to produce recombinant viral gene therapy vectors [87,88,89] and virus-like particle-based vaccines [90,91]. Furthermore, researchers have shown efficient expression of recombinant proteins in microalgae using a geminiviral vector as a proof-of-concept method to produce recombinant proteins quickly and at scale [92].

Despite the high efficiency and low cost of some non-mammalian expression systems, there exist limitations. Notably, species-specific differences in glycosylation, protein-folding processes that require special chaperones, and post-translational modifications can lead the products of mammalian cell-based expression systems to have considerable advantages for therapeutic applications over proteins produced in non-mammalian cell expression systems [93,94]. Multiple studies have shown that therapeutic AAVs produced using baculovirus and Sf9 cells can be physically distinct and transduce target cells at lower efficiencies than AAVs produced in human embryonic kidney (HEK293) production systems [95,96,97].

In addition to using whole DNA viruses as protein expression vectors, promoters originating from DNA viruses are routinely incorporated into plasmids or cellular genomes to achieve strong expression of proteins. As a ubiquitous example, a promoter from the T7 bacteriophage is used with the T7 RNA polymerase to express proteins in a wide variety of prokaryotic and even eukaryotic protein expression systems [98,99]. Likewise, promoters from a type of herpesvirus known as cytomegalovirus (CMV) [100] and a polyomavirus known as Simian Virus 40 (SV40) [101] are commonly used in both viral and nonviral vectors to drive protein expression using mammalian gene expression machinery. To illustrate the widespread use of these promoters derived from DNA viruses, a search on www.addgene.org on 12 July 2023 returned 25,525 plasmids for the keyword “CMV”, 8308 plasmids for the keyword “T7”, and “2318” plasmids for the keyword “SV40”. Nonetheless, viral-derived promoters such as CMV are prone to epigenetic silencing in mammalian cells. As such, efforts to engineer promoters with more durable and controllable expression are underway.

Biomanufacturing proteins using molecular machinery derived from DNA viruses has aided basic and translational research for over 50 years. With further discovery and optimization, gene expression tools derived from DNA viruses will continue to advance biomanufacturing.

### 2.5. Mitigating Antibiotic Resistance

Bacterial pathogens harboring resistance to multiple classes of antibiotic drugs pose a serious and growing threat to public health [102]. DNA phage viruses show promise to treat bacterial infections and slow the spread of drug resistance. As with the use of viruses to treat cancer, the idea of “phage therapy” has been around for over 100 years. Shortly following the discovery of phage viruses in the late 1910s by Frederik Twort and Félix d’Hérelle, d’Hérelle recorded successful examples of using phage viruses to treat laboratory chickens infected with *Salmonella gallinarum* and human dysentery patients naturally infected with *Shigella dysenteriae* [103,104]. Nevertheless, phage therapy has faced multiple limitations, including insufficient knowledge to match phage cultures with bacterial species underlying infections, the tendency of bacteria to rapidly evolve resistance to phages with narrow host ranges, and poor biodistribution following in vivo delivery. The discovery of Penicillin by Alexander Fleming in 1928 resulted in widespread reliance on chemical-based antibiotics accompanied by generally decreased interest in phage therapy [105]. However, the stalling development of new chemical-based antibiotic drug classes has necessitated the discovery and commercialization of mechanistically novel treatments for bacterial infections, leading to a resurgence in phage therapy.

Phage therapy in the age of genetic engineering boasts numerous advantages, including its engineerable tropism to specific pathogenic bacterial strains, lowered off-target effects in human cells relative to chemical-based antibiotics, reduced off-target killing of helpful bacterial populations in the microbiome, and potential synergy to boost the efficacy and reduce the risk of bacterial resistance to conventional antibiotics. Accordingly, phage therapy is a resurging area of research as evidenced by the 18 active or recruiting clinical trials as of January 2025 [61], the publication of multiple clinical studies and review articles [106,107,108,109,110,111,112,113,114], an increasing number of institutional centers dedicated to phage therapy research [115,116,117,118,119], and active funding initiatives by governmental agencies [119]. An example of recent technological progress includes a method for continuous and multiplexable phage genome modifications using a modified bacterial recombitron that does not require counterselection, which enables the concurrent editing of multiple distinct phages and incorporation of five different mutations in phage lambda and thereby paves the way for improved scalability of phage therapies [120]. In addition to their potential use as therapeutic products, some DNA phage viruses have been used as engineering platforms to create better complex proteins. For example, a phage virus was recently used to optimize prime editors—an approach that can enable in vivo DNA editing without generating double-stranded breaks [121].

As more is learned about the underlying mechanisms of phage replication cycles, phage/bacteria interactions, and complex microbial ecosystems within the human body, DNA phage viruses may rise as valuable treatments and preventative measures to promote human health.

### 2.6. Editing Microbiomes

The essential role of the microbiome in human health and disease has been increasingly understood and appreciated in recent years. Compelling evidence (extensively reviewed in the cited articles) now exists linking dysbiosis of microbial communities with cancerous [122,123,124], autoimmune [125,126,127,128], infectious [129,130,131], metabolic [132,133], cardiovascular [134,135], and neurological [136,137,138,139,140] diseases. Accordingly, the idea of engineering the microbiome to restore health has gained traction in both academia and the biotechnology industry. As of June 2023, dozens of research centers are dedicated to understanding and modulating the microbiome, including the University of California’s Berkeley Initiative for Optimized Microbiome Editing (BIOME), which seeks to precisely edit microbial communities in their natural environments using CRISPR technology [141].

The microbiome consists of complex networks of remarkably diverse bacteria, rendering the genetic engineering of microbiomes particularly challenging from a delivery perspective. Though alternative delivery approaches have been reported [142,143,144,145,146], phage-mediated delivery is currently ideal for applications that seek to target a specific species or group of bacteria without affecting off-target species in complex microbial communities. Pioneering studies have provided proof-of-concept for the use of temperate phages containing DNA genomes as useful tools in microbiome engineering. For example, an engineered programmable dCas9-expressing bacteriophage λ to repress the *stx2* gene encoding Shiga toxin of *E. coli* in the gut [147] and reduce Shiga toxin production [148] in murine models has been reported. Lam and colleagues demonstrated the capability of engineered bacteriophage M13 to deliver a programmable, exogenous CRISPR-Cas9 system for strain-specific targeting in the murine gastrointestinal tract [149]. Another group utilized bacteriophages T7 and λ to deliver CRISPR base editors to a synthetic soil microbial community [150]. In yet another example, cytolysin—a two-subunit endotoxin secreted by *E. faecalis*—was shown to be implicated in alcohol hepatitis and successfully targeted human-derived *E. faecalis* using strain-specific phage viruses in humanized mouse models [151]. Recently, multiple genes of *E. coli* were edited in situ using phage-derived particles delivering a base editor, achieving 93% editing efficiency with one dose of the phage particles [152]. These data demonstrate the potential to directly edit the gut microbiome using phage particles and expand the ability to design new microbiome-targeted therapies.

One historical limitation in the application of viral gene delivery vectors to microbiome editing is the necessity to propagate phages in their target bacteria after using traditional transformation techniques to synthesize them. Phages can potentially evolve altered host range during scale-up, which could affect their efficiency in the clinic. To mitigate this challenge, cell-free transcription-translation (TXTL) has been used to produce therapeutic phages, which may help facilitate expanded phage production for clinical use in the future [109,153]. Given the vast complexity of the microbiome, identifying which microbial target species and cargoes will have a therapeutic effect is also a challenge. Advances in artificial intelligence (AI), next-generation sequencing (NGS), and directed evolution technologies will aid in decoding the complexity of microbiomes, identifying bacterial species and gene-specific targets, and generating effective phage-based gene delivery systems to promote human health.

### 2.7. Agriculture

The utility of DNA viruses as biotechnological tools transcends research and health; DNA viruses have also been developed for agricultural and pest control applications. One historical example of their use as a pest control agent was the release of myxoma virus (MYXV) in 1950 to control the invasive European rabbit in Australia [154]. As a member of the *Poxviridae* family and *Leporipoxvirus* genus, MYXV’s host range is tightly restricted to rabbits [14]. European rabbits had been brought to Australia by settlers from Europe but subsequently multiplied in the absence of natural predators. Profound destruction to Australia’s agricultural and natural ecological systems ensued. While MYXV is a natural virus endemic to new-world rabbits, European rabbits lacked prior immunological exposure. Within several years of its release on the Australian continent, MYXV wiped out nearly 100% of the European rabbits with no host spillover events outside of rabbits and hares. While hailed as a success, a small number of rabbits harbored random mutations that conferred resistance to MYXV. A host–pathogen evolutionary arms race ensued, with the European rabbits evolving immunological mechanisms to overcome the virus while the virus evolved strategies to antagonize the immune system of the host [155,156,157]. Seven years following its introduction in Australia, the mortality rate of MYXV fell to less than 30% in field rabbits on the continent. That said, today MYXV is endemic in rabbit populations of Australia, and the populations of European rabbits in Australia have never reached their pre-1950 levels [158].

DNA viruses are also utilized for insect pest control. In many ecosystems, DNA viruses are known to naturally regulate insect populations [159,160,161,162]. Baculoviruses, for example, are harmless to humans and ubiquitously found in many vegetables common in the human diet. The host range of most baculoviruses is tightly restricted to individual insect species or closely related groups of insects [163]. Thus, baculoviruses are in general thought to be safer alternatives to chemical-based pest control from both environmental and health standpoints. Observations of the role of baculoviruses in natural ecosystems and their safety in humans led to their commercial application to protect agricultural crops from insect pests. Wild-type and recombinant baculoviruses have shown success in managing a number of harmful pest populations, including the larvae of *Galleria mellonella*, which destroy honeybee hives; *Phthorimaea operculella*, which destroy potatoes, *Spodoptera littoralis*, which destroy cotton; and Lepidopteran and Hymenopteran forest pests [164,165,166,167,168]. Additionally, densoviruses have shown potential as agents to control mosquito populations and reduce the transmission of dengue viruses to humans [169,170,171,172,173]. As of January 2025, there are 7 DNA virus-based biopesticide formulations registered for commercial use by the US Environmental Protection Agency (EPA) [174] and others approved globally (Table 4).

DNA viruses have also shown potential as gene delivery vectors for genetically engineered agricultural crops and livestock. Genetic engineering has great potential to improve production yields; resistance to drought, heat, pathogens, and pests; and the nutritional value of food. As with any genetic engineering approach, effective delivery of gene editing machinery is crucial. AdVs, herpesviruses, and modified vaccinia Ankara (MVA) have been used in food animals as vectored vaccines [175,176,177,178,179,180] or to deliver gene-editing machinery to target cells [181,182]. Furthermore, a group of plant viruses containing circular ssDNA genomes known as Geminiviruses have proven to be useful for genetically engineering plants. For example, the bean yellow dwarf virus (BeYDV) and cabbage leaf curl virus (CaLCuV) have delivered zinc finger nuclease (ZFN), transcription activator-like effector nucleases (TALEN), CRISPR-Cas9 machinery, and homology-directed repair (HDR) templates to wheat, tobacco, tomato, or potato plants [183,184,185,186,187,188,189].

Nonetheless, hurdles remain for applying DNA viruses to agricultural applications. These include (1) manufacturing large quantities of virus; (2) in the case of biopesticides, administering virus suspensions in a manner that promotes ingestion by target pests; (3) overcoming the tendency of pests to rapidly evolve resistance; and (4) achieving delivery and cargo expression specificity in the target host species without spillover into native and non-pest species. Further research to increase our understanding of the molecular mechanisms involved in large DNA virus replication cycles, improve manufacturability, and develop cargoes that confer strong efficiency and specificity will enable large DNA viruses to add immense value to sustainably feeding the growing human population.

## 3. Discussion

### 3.1. Biosafety

Advancing techniques to engineer viruses comes with the potential to do transformative good or catastrophic harm, depending on how the technology is applied. As such, biosafety must be upheld as the highest priority in the application of viruses to biotechnology, while at the same time ensuring that public policies do not prevent the rapid advancement of virology knowledge, vaccines, and therapeutics, which are central to society’s ability to fight and prevent genetic and infectious diseases. For example, the robustness and accessibility of virology research enabled the rapid development of safe and effective vaccines and antivirals during the COVID-19 pandemic [190,191]. At the same time, questions about the origin of SARS-CoV-2 prompted pushes to strengthen regulations on virology research [192]. Overly restrictive regulation of virology research could hinder research and development on viral vectors that pose no reasonable risk to the public but are instead crucial to the development of new vaccines and therapeutics for diseases of unmet need. Accordingly, numerous experts have advocated for the oversight of pathogen research to be carefully calibrated and clearly defined [193].

### 3.2. Future Perspectives

DNA virus vectors have demonstrated the ability to bring immense environmental, agricultural, and health benefits to humankind. While previously reported engineering techniques have led to improved vectors for gene delivery and expression, new advances in disease modeling, machine learning, NGS, gene modification, DNA synthesis, and DNA diversification technologies will further unlock the powerful potential of DNA virus vectors to deliver solutions to current and future challenges. As viral engineering technologies progress, responsible measures must be taken in parallel to mitigate the risk of misuse without stifling crucially needed scientific progress.

## Figures and Tables

**Table 1:** Clinically approved DNA virus-based vaccine products.

Name	DNA Virus	Indication(s)	First Approval
VARIVAX (Varicella Virus Vaccine, Live)	^7^ VZV	Chickenpox/shingles	17 March 1995 (^2^ US FDA)
PROQUAD (Measles, Mumps, Rubella and Varicella Virus Vaccine Live)	^7^ VZV (+RNA Viruses)	Chickenpox/shingles	6 September 2005 (^2^ US FDA)
ACAM2000 (Smallpox and Mpox Vaccine, Live)	^8^ VACV	Smallpox and mpox	31 August 2007 (^2^ US FDA)
ZOSTAVAX (Zoster Vaccine, Live)	^7^ VZV	Singles	25 May 2006 (^2^ US FDA)
Adenovirus Type 4 and Type 7 Vaccine, Live, Oral	^9^ AdV	Febrile acute respiratory disease caused by Adenovirus Type 4 and Type 7	16 March 2011 (^2^ US FDA)
PRUIORIX TETRA (monovalent and multivalent measles, mumps, rubella and varicella vaccines)	^7^ VZV	Chickenpox/shingles	27 June 2013 (^3^ Germany PEI)
IMVANEX (smallpox and monkeypox vaccine)	^10^ MVA	Smallpox and mpox	31 July 2013 (^4^ EMA)
JYNNEOS (Smallpox and Mpox Vaccine, Live, Non-replicating)	^10^ MVA	Smallpox and mpox	24 September 2019 (^2^ US FDA)
PROVARIX (varicella vaccine, live)	^7^ VZV	Chickenpox/shingles	18 December 2019 (^5^ China NMPA)
^1^ AstraZeneca COVID-19 Vaccine (ChAdOx1 nCoV-19/AZD1222)	^9^ AdV	^11^ COVID-19	20 December 2020 (^6^ UK MHRA)
^1^ Janssen COVID-19 Vaccine (Ad26.COV2.S)	^9^ AdV	^11^ COVID-19	27 February 2021 (^2^ US FDA)

^1^ Approved under an emergency use scenario. ^2^ United States Food & Drug Administration. ^3^ Paul Ehrlic Institut, also known as the Federal Institute for Vaccines and Biomedicines. ^4^ European Medicines Agency. ^5^ China National Medical Products Administration. ^6^ United Kingdom Medicines and Healthcare Products Regulatory Agency. ^7^ Varicella zoster virus. ^8^ Vaccinia virus. ^9^ Adenovirus. ^10^ MVA = Modified vaccinia Ankara. ^11^ Coronavirus Disease 2019.

**Table 2:** Clinically approved DNA virus-based gene therapy products.

Name	Vector	Indication(s)	First Approval
^1^ alipogene tiparvovec (GLYBERA)	^4^ AAV1	familial lipoprotein lipase deficiency (LPLD)	2 November 2012 (^3^ EMA)
voretigene neparvovec-rzyl (LUXTURNA)	^4^ AAV2	Biallelic RPE65 mutation-association retinal dystrophy	18 December 2017 (^2^ US FDA)
onasemnogene abeparvovec-xioi (ZOLGENSMA)	^4^ AAV9	Spinal muscular atrophy	24 May 2019 (^2^ US FDA)
eladocagene exuparvovec-tneq (KEBILIDI/ UPSTAZA)	^4^ AAV2	Aromatic L amino acid decarboxylase (AADC) deficiency	18 July 2022 (^3^ EMA)
valoctocogene roxaparvovec-rvox (ROCTAVIAN)	^4^ AAV5	Hemophilia A	24 August 2022 (^3^ EMA)
etranacogene dezaparvovec-drlb (HEMGENIX)	^4^ AAV5	Hemophilia B	22 November 2022 (^2^ US FDA)
beremagene geperpavec (VYJUVEK)	^5^ HSV-1	Dystrophic epidermolysis bullosa	19 May 2023 (^2^ US FDA)
delandistrogene moxeparvovec-rokl (ELEVIDYS)	^6^ AAVRh74	Duchenne muscular dystrophy	21 June 2023 (^2^ US FDA)
fidanacogene elaparvovec-dzkt (BEQVEZ)	^7^ AAVRh74var	Hemophilia B	25 April 2024 (^2^ US FDA)

^1^ Subsequently withdrawn due to lack of demand. ^2^ United States Food & Drug Administration. ^3^ European Union European Medicines Agency. ^4^ Adeno-associated virus. ^5^ Herpes simplex virus. ^6^ Adeno-associated virus Rhesus serotype 74. ^7^ Adeno-associated virus Rhesus serotype 74 variant.

**Table 3:** Clinically approved DNA virus-based oncolytic virotherapy products.

Name	Vector	Indication(s)	First Approval
^1^ rAd-p53 (GENDICINE)	^5^ AdV	Head and neck squamous cell carcinoma (HNSCC)	October 2003 (China ^2^ SFDA)
H101 (ONCORINE)	^5^ AdV	Head and neck and esophagus cancer, Nasopharyngeal cancer	1 November 2005 (China ^2^ SFDA)
talimogene laherparepvec (T-VEC, IMLYGIC)	^6^ HSV	Melanoma	27 October 2015 (^3^ US FDA)
Teserpaturev (DELYTACT)	^6^ HSV	Malignant glioma	June 2021 (Japan ^4^ PMDA)
^1^ nadofaragene firadenovec-vncg (ADSTILADRIN)	^5^ AdV	Bacillus Calmette-Guérin (BCG)-unresponsive non-muscle invasive bladder cancer (NMIBC) with carcinoma in situ (CIS)	16 December 2022 (^3^ US FDA)

^1^ Replication-incompetent. ^2^ State Food and Drug Administration, subsequently renamed to National Medical Products Administration. ^3^ United States Food and Drug Administration. ^4^ Pharmaceuticals and Medical Devices Agency. ^5^ Adenovirus. ^6^ Herpes simplex virus.

**Table 4:** Recently approved ^1^ DNA virus-based biopesticides.

Name	Active Ingredient	First Approval
Multiple Products	^6^ AcMNPV	Unspecified (^3^ EC)
Multiple Products	^7^ SeMNPV	17 May 2007 (^3^ EC)
Multiple Products	^8^ CpGV	1 May 2009 (^3^ EC)
VIROSOFT™CP4	^8^ CpGV	16 July 2010 (^4^ US EPA)
CYD-X^®^ PLUS/CYD-X^®^ HP	^8^ CpGV	21 July 2011 (^4^ US EPA)
Multiple Products (e.g., HELICOVEX^®^)	^9^ HearNPV	22 April 2013 (^3^ EC)
Heligen/Armigen/ Armigen Vivus/Vivus Max	^10^ HzNPV strain ABA-NPV-U	5 March 2014 (^4^ US EPA)
SPEXIT^®^	^7^ SeMNPV strain BV-0004	2 December 2015 (^4^ US EPA)
SURTIVO^®^/ Surtivo Soy	^10^ HzNPV strain ABA-NPV-U	20 March 2020 (^4^ US EPA)
^5^ PD20230095	^11^ AfMNPV Kew1	23 March 2023 (^2^ China MARA)
Multiple Products (e.g., PD20230100)	^12^ SfMNPV Hub1	23 March 2023 (^2^ China MARA)
^5^ PD20230093	^11^ AfMNPV Kew1	23 March 2023 (^2^ China MARA)
Multiple Products	Betabaculovirus phoperculellae	22 January 2025 (^3^ EC)

^1^ List includes examples of products first approved between January 2005 and January 2025. ^2^ China Ministry of Agricultural and Rural Affairs. ^3^ European Commission. ^4^ United States Environmental Protection Agency. ^5^ Indicates a governmental registration number. ^6^ *Autographa californica* nucleopolyhedrovirus. ^7^ *Spodoptera exigua* nucleopolyhedrovirus. ^8^ *Cydia pomonella* granulovirus. ^9^ *Helicoverpa armigera* nucleopolyhedrovirus. ^10^ *Helicoverpa zea* Nucleopolyhedrovirus. ^11^ *Anagrapha falcifera* nucleopolyhedrovirus. ^12^ *Spodoptera frugiperda* multiple nucleopolyhedrovirus.

## Data Availability

No new data were created or analyzed in this study. Data sharing is not applicable to this article.

## Abbreviations

The following abbreviations are used in this manuscript:

AAV	Adeno-Associated Virus
AdV	Adenovirus
AI	Artificial Intelligence
BeYDV	Bean Yellow Dwarf Virus
BIOME	Berkeley Initiative for Optimized Microbiome Editing
CaLCuV	Cabbage Leaf Curl Virus
COVID-19	Coronavirus Disease 2019
CRISPR	Clustered Regularly Interspaced Short Palindromic Repeats
DNA	Deoxyribonucleic Acid
EC	European Commission
EMA	European Medicines Agency
EPA	Environmental Protection Agency
EU	European Union
FDA	Food and Drug Administration
HLA	Human Leukocyte Antigen
HSV	Herpes Simplex Virus
MARA	Ministry of Agricultural and Rural Affairs
MHC	Major Histocompatibility Complex
MHPRA	Medicines and Healthcare Products Regulatory Agency
MVA	Modified Vaccinia Ankara
MYXV	Myxoma Virus
NGS	Next-Generation Sequencing
NMPA	National Medical Products Administration
PEI	Paul Ehrlic Institut
RNA	Ribonucleic Acid
SARS-CoV-2	Severe Acute Respiratory Syndrome Coronavirus 2
SFDA	State Food and Drug Administration
TALEN	transcription activator-like effector nucleases
TXTL	cell-free transcription-translation
UK	United Kingdom
US	United States
VACV	Vaccinia Virus
VZV	Varicella Zoster Virus
ZFN	Zinc finger nuclease
